# Supplementary material for: The role of CO2 in the genesis of Dabie-type porphyry molybdenum deposits
Source: Nat Commun. 2024 Jun 6;15:4849. doi: 10.1038/s41467-024-49275-0 (PMC11156875; doi:10.1038/s41467-024-49275-0)
Supplement: Supplementary file 1 — Supplementary Information [file 41467_2024_49275_MOESM1_ESM.pdf]

Supplemental material: Additional results, modeling and the tectonic setting of Dabie-type Mo deposits.

Zi-Qi Jiang[1], Lin-Bo Shang[1*], A. E. Williams-Jones[2], Xin-Song Wang[1*], Li Zhang[3], Huai-Wei Ni[3], Rui-Zhong Hu[1], and Xian-Wu Bi[1]

[1]State Key Laboratory of Ore Deposits Geochemistry, Institute of Geochemistry, Chinese Academy of Sciences, Guiyang 550081, China

[2]Department of Earth and Planetary Sciences, McGill University, 3450 University Street, Montreal, QC H3A 0E8, Canada

[3]CAS Key Laboratory of Crust-Mantle Materials and Environments, School of Earth and Space Sciences, University of Science and Technology of China, Hefei 230026, China

Corresponding Author:

*E-mail: shanglinbo@vip.gyig.ac.cn;

*E-mail: wangxinsong@mail.gyig.ac.cn

Contents:

S1. Additional results

S2. Comparison of D_{Mo} values to published values

S3. Modeling the efficiency of molybdenum extraction from magmatic fluids

S4. The nature of the tectonism and magmatism associated with Dabie-type Mo deposits

S1. Additional results

1.1 The concentration of Mo in equilibrium fluids and quenched felsic glasses.

As mentioned in the method section, fluid-melt partitioning experiments were conducted in the systems H_2O-CO_2, H_2O-NaCl and H_2O-NaCl-CO_2 at 850 °C and 100 or 200 MPa. In the system H_2O-CO_2, X_{CO2} was varied between 0.10 and 0.20 and in experiments in the system H_2O-NaCl, the proportion of NaCl was varied between 1.1 wt.% and 44 wt.%. The value of X_{CO2} and the proportion of NaCl in the system H_2O-NaCl-CO_2 varied from 0.11 to 0.31 and 2.2 to 3.1 wt.% in the brine and vapor, respectively, and in the brine, the proportion of NaCl was between 56 and 62 wt.%. The

experiments all had a duration of 10-12 days. To establish whether this was sufficient to reach equilibrium, i.e., for any gradients in Mo concentration in the melt to be eliminated, the concentration of Mo and the major elements was analyzed along traverses from one edge of pieces of the quenched glasses to the other edge (Supplementary Table 4). The results show that there was no gradient in element concentration, that the relative standard deviation for the concentration of Mo was less than 20% and that, in the case of the major elements, it was less than 10%. This relatively homogeneous distribution of the elements in the glass shows that equilibrium was reached during the experiments.

The major element composition of the quenched glasses was generally very similar to that of the starting material. In experiments involving H_2O-NaCl and H_2O-NaCl-CO_2 fluids, however, the concentration of K_2O in the glasses displayed a small but progressive decrease with increasing salinity and CO_2 content. This decrease was $\leq$1 wt.%, except in experiment KD-003, in which the fluid had an initial salinity of 42.6 wt.%; the K_2O content of the quenched glass in this experiment decreased from 5.95 to 1.36 wt.%. Despite this decrease in K_2O content, the A/NK (mole ratio of Al_2O_3 and Na_2O+K_2O of the quenched glasses) value of the melt in experiments involving H_2O-CO_2 and H_2O-NaCl fluids remained unchanged. However, the A/NK ratio of the melt in experiments involving H_2O-NaCl-CO_2 fluids decreased slightly compared to that of the starting material due to a decrease in the Al_2O_3 content of the quenched glass.

The glasses from experiments with fluids in the H_2O-CO_2 system all had very similar Mo contents of ~ 400 ppm, despite the variation in X_{CO2}. However, the concentration of Mo in the quenched glasses from the experiments with fluids in the H_2O-NaCl system decreased with increasing fluid salinity, and in the experiments involving H_2O-NaCl-CO_2 fluids, it decreased with both increasing salinity and increasing CO_2 content. The concentration of Mo in the quenched glass varied from 359 to 13.6 and 189 to 112 ppm, respectively, for the two fluid systems (Supplementary Figure. 4; Supplementary Table 5).

The Mo concentration of the reacted fluids in the H_2O-CO_2 system varied little and unsystematically with respect to X_{CO2}. It ranged from 82 ppm to 117 ppm for X_{CO2} values between 0.1 and 0.2.

In the H_2O-NaCl system, the concentration of Mo in this fluid increased with increasing salinity of the fluid from ~180 ppm for a salinity of 1.1 wt.% NaCl to ~430 ppm for a salinity of 21 wt.% NaCl. The concentrations of Mo obtained by the leaching and fluid inclusion methods (Supplementary materials 1.3) were indistinguishable within the experimental and analytical uncertainty of the two methods (Supplementary Figure 2a).

Brine and vapor were trapped as separate phases in the H_2O-NaCl-CO_2 system, as shown by the presence of vapor and brine inclusions, as well as heterogeneously (variable proportions of vapor and brine), in quartz from the experiments for this system (Fig. 1). To ensure that inclusions containing heterogeneously entrapped liquid and vapor were not considered in our evaluation of the composition of the vapor and brine inclusions and in determining D_{Mo}, we only measured the compositions of fluid inclusions in fluid inclusion assemblages for which the phase ratios were consistent[1]. As a result of the phase separation, the salinity of the brine increased from that of the homogeneous system (~7 wt.%) to 56 wt.% NaCl at a X_{CO2} of 0.1 and 62 wt.% NaCl at a X_{CO2} of 0.3. The corresponding maximum concentrations of Mo in the brine were 11,452 ppm and ~20,000 ppm (KD-005 and Q-017). Because of the large bubble size of the vapor inclusions (>80% of the inclusion volume) (Fig. 1c), there was a large variation in the clathrate melting temperature and a correspondingly large uncertainty in the estimated salinity of the vapor inclusions (Supplementary Table 3). The average salinity of these inclusions was ~ 3.1 wt.% NaCl for a X_{CO2} of ~0.1 and 2.2 wt.% NaCl for a X_{CO2} of 0.31 and the concentration of Mo was ~ 400 ppm.

1.2 The fluid-melt partition coefficient for Mo ($D_{Mo}^{fluid/melt}$)

The data for $D_{Mo}^{fluid/melt}$ obtained in this study are summarized in Supplementary Table 5 and the specific results are reported in the results section of this paper.

1.3 The influence of salinity on D_{Mo}

Although the $D_{Mo}^{fluid/melt}$ values increase with increasing salinity in the lower

salinity part (≤ 20 wt.%) of the H_2O-NaCl system, this increase is relatively small (0.7 to 3.2). In contrast, in the high salinity part (≥ 20 wt.%) of the H_2O-NaCl system, the increase in the $D_{Mo}^{fluid/melt}$ value is large, reaching 25.7 at a salinity of 44.3 wt.% NaCl, i.e., the variation of $D_{Mo}^{fluid/melt}$ with salinity is exponential. Thus, hypersaline fluids are very efficient in leaching Mo from coexisting felsic melts. Indeed, in the brine-vapor-melt system, the $D_{Mo}^{brine/melt}$ value reached 179 at a salinity of 62 wt.% NaCl (X_{CO2} = 0.3). Thus, based on the Mo concentration of the melt employed in our experiments, the solubility of Mo in such a brine could reach ~ 20,000 ppm. We are not implying, however, that such a high concentration might be realized in nature as the Mo concentrations of the magmas are likely to be much lower than those of the melt employed in our experiments.

S2. Comparison of D_{Mo} values to published values

Because the value of $D_{Mo}^{fluid/melt}$ is a function of a number of parameters, notably pressure, temperature, fO_2, fS_2, and the melt and fluid composition[2–8], it is difficult to reliably compare the values of $D_{Mo}^{fluid/melt}$ obtained in the different studies, including ours. Several studies, however, have reported trends of $D_{Mo}^{fluid/melt}$ with salinity similar to that reported here[4–7]. In all cases the $D_{Mo}^{fluid/melt}$ value for the high salinity fluids was reported to be ≥ 100 times higher than that for the low salinity fluids (Fig.3). The values reported by Tattitch and Blundy[5], and Fang and Audetat[6] are consistently higher than those of the current study. A possible reason for this is the presence of sulfur in their experiments, which is interpreted by the the authors of these papers to increase the solubility of Mo in the fluids relative to that of Mo in sulfur-free experiments (Fig.3).

S3. Modeling the efficiency of molybdenum extraction from magmatic fluids

The efficiency of molybdenum extraction from magmatic fluids is defined as the amount of Mo in the fluid exsolved from the magma divided by the amount of molybdenum initially in the magma. In calculating this efficiency of extraction, a hypothetical chamber containing 100 km^3 of magma with a concentration of 15 ppm Mo[9], a water content of 5 wt.%, and a density of 2.5 g/cm^3 was assumed. The molybdenum in the hydrothermal fluid was assumed to have a 100% precipitation efficiency and the mass proportion of fluid exsolved from the magma was assumed to

be 10%. Because the fluids in the H_2O-NaCl-CO_2 system were in the two-phase region under the experimental conditions, the brine and the vapor could be considered to represent the exsolution of fluid from a magma with the same composition as the melt. Based on the initial salinity of the H_2O-NaCl-CO_2 fluid, microthermometric estimates of brine and vapor salinity and the lever rule, the mass ratio of brine and vapor was determined to have been between 0.07 and 0.1. A mass ratio of 0.1 was used in the calculation of extraction efficiency. Accordingly, the bulk partition coefficient ($D_{Mo}^{bulk} = D_{Mo}^{brine} * 1/11 + D_{Mo}^{vapor} * 10/11$) was recalculated in the H_2O-NaCl-CO_2 system based on this assumption (details of the calculation are provided in Supplementary Table 6). As the experiments for the H_2O-NaCl-CO_2 fluid-melt system employed a peralkaline rather than a peraluminous composition and D_{Mo} values for the latter melt composition are approximately half those for peralkaline melts, the D_{Mo} values were assumed to be half those measured. The H_2O-NaCl fluid, however, was in the supercritical region under the experimental conditions and the $D_{Mo}^{fluid/melt}$ values could be determined directly from the measured concentrations of Mo in the fluid and the glass. In order to evaluate the role of CO_2 in improving the efficiency of extracting Mo from magma, the $D_{Mo}^{brine/melt}$ value for the H_2O-NaCl-CO_2 system was determined for a similar bulk salinity to that of the supercritical fluid in the H_2O-NaCl system. The starting salinity of the H_2O-NaCl-CO_2 system is ~ 7 wt.%, and the D_{Mo} obtained for a salinity of 6.3 wt.% of H_2O-NaCl system was, therefore, used for calculation. The equation of $M_{Mo}^{residual\ magma}=M_{Mo}^{initial\ magma}*F^{D}$ was used to calculate the mass of Mo in the residual magma, where F represents the fraction of the exsolved fluids and D represents the partition coefficient. The extraction efficiency was defined as the mass of Mo extracted by the exsolved fluids divided by the mass of Mo in the initial magma.

S4. The nature of the tectonism and magmatism associated with Dabie-type Mo deposits

The Dabie-type porphyry molybdenum deposits developed in a post-collisional extensional environment. The tectonic change from collisional compression to extension was associated with collapse, delamination, and thinning of over-thickened

orogenic crust and lithosphere, accommodating large-scale magmatism and mineralization[10,11]. As a result, the granitic rocks related to ore-formation are metaluminous to peraluminous, and high-K calc-alkaline to shoshonitic. In addition, the Sr/Y ratios of these crust-derived granites decrease with their age from ~140 Ma to <127 Ma, reflecting thinning from over-thickened crust to crust of normal thickness (<35 km) in the area[10–12]. The carbon isotope compositions of the fluid inclusions are in the range from -2.3 ‰ to +2.7 ‰[13] suggesting that the CO_2 of the magmas originated from a mixture of recycled carbonate rocks and mantle. Rare Au/Cu mineralization is associated with the Mo mineralization.

Supplementary Table 1. The initial composition of the glasses used in the experiments.

T °C	P MPa	Na_2O, wt.%	Al_2O_3,wt.%	SiO_2, wt.%	K_2O, wt.%	A/NK	Mo, ppm
CO_2-H_2O series							
850	100	5.28	11.1	77.1	6.41	0.71	592
NaCl-H_2O series							
850	200	3.92	12.4	77.6	5.95	0.96	656
NaCl-CO_2-H_2O series							
850	200	5.28	11.1	77.1	6.41	0.71	592

Supplementary Table 2. Initial fluid composition.

No.	Salinity, wt.%	XCO_2, mol%
CO_2-H_2O-series		
C-002	N.D.	0.1
C-003	N.D.	0.15
C-005	N.D.	0.15
C-006	N.D.	0.2
NaCl-H_2O series		
Q-004	4.93	N.D.
Q-008	9.92	N.D.
Q-001	6.94	N.D.
Q-006	1.97	N.D.
H-002	6.94	N.D.
KD-001	14.8	N.D.
KD-002	19.8	N.D.
KD-003	42.6	N.D.
NaCl-CO_2-H_2O series*		
Q-011	7.4	0.11
Q-014	7.8	0.15
Q-016	7.3	0.21
Q-017	7.1	0.31
KD-005	7.4	0.30

Note:

N.D. = no data.

*As the decomposition of oxalic acid produces water, solid NaCl was added to ensure that the salinity of the starting solution in the NaCl-CO_2-H_2O series experiments corresponded to that selected.

Supplementary Table 3. Microthermometric measurements.

	XCO_2	Decomposition temperature of clathrate, ℃	Salinity of the vapor, wt.%	Dissolution temperature of halite, ℃	Salinity of the brine, wt.%	Melting temperature of ice, ℃	Salinity of the supercritical fluid, wt.%
Q-011	0.11	8.5-8.9	3.0-2.2	472	56	N.D.	N.D.
Q-014	0.15	N.A.	N.A.	490	58	N.D.	N.D.
Q-016	0.21	8.2-8.6	3.5-2.8	494	59	N.D.	N.D.
Q-017	0.31	N.A.	N.A.	517	62	N.D.	N.D.
KD-005	0.30	8.7-9	2.6-1.8	515	62	N.D.	N.D.
KD-001	N.D.	N.D.	N.D.	N.D.	N.D.	-12.7	17
KD-002	N.D.	N.D.	N.D.	N.D.	N.D.	-18.3	21
KD-003	N.D.	N.D.	N.D.	370	N.D.	N.D.	44
Q-006	N.D.	N.D.	N.D.	N.D.	N.D.	-0.6	1.1
Q-004	N.D.	N.D.	N.D.	N.D.	N.D.	-2.3	3.9
Q-001	N.D.	N.D.	N.D.	N.D.	N.D.	-3.9	6.3
Q-008	N.D.	N.D.	N.D.	N.D.	N.D.	-7.2	11
H-002	N.D.	N.D.	N.D.	N.D.	N.D.	-4.1	6.6

Note:

N.D.- no data.

N.A.- the data are not applicable because of the large uncertainty.

Supplementary Table 4 Major element composition of the quenched glass in wt%, and Mo concentration in ppm.

No.	N	Na_2O	σ	RSD	Al_2O_3	σ	RSD	SiO_2	σ	RSD	K_2O	σ	RSD	A/NK	Mo	σ	RSD
H_2O-CO_2 experiments																	
C-002	6	5.14	0.04	0.78	10.9	0.19	1.74	77.5	0.23	0.30	6.42	0.02	0.31	0.71	401	2.06	0.51
C-003	8	5.13	0.02	0.39	10.8	0.06	0.56	77.6	0.04	0.05	6.44	0.01	0.16	0.7	474	4.13	0.87
C-005	8	5.13	0.06	1.17	10.8	0.21	1.94	77.6	0.32	0.41	6.48	0.06	0.93	0.7	456	6.68	1.46
C-006	8	4.82	0.09	1.87	10.4	0.33	3.17	78.6	0.47	0.60	6.18	0.08	1.29	0.71	424	3.88	0.92
NaCl-H_2O experiments																	
Q-004	8	3.53	0.22	6.23	11.7	0.62	5.30	78.8	0.93	1.18	5.94	0.58	9.76	0.95	277	14.9	5.38
Q-008	7	4.46	0.16	3.59	11.2	0.54	4.82	79.9	0.79	0.99	4.48	0.1	2.23	0.92	162	28.4	17.5
Q-001	8	4.7	0.13	2.77	11.2	0.27	2.41	79.8	0.47	0.59	4.3	0.47	10.9	0.9	228	18.3	8.03
Q-006	7	5.09	0.34	6.68	12.9	0.74	5.74	78.0	1.34	1.72	3.97	0.15	3.78	1.02	359	83.4	23.2
H-002	7	4.81	0.07	1.46	11.6	0.21	1.81	79.3	0.29	0.37	4.3	0.04	0.93	0.93	205	35.9	17.5
KD-001	10	5.53	0.07	1.27	11.7	0.28	2.39	79.6	0.35	0.44	3.17	0.09	2.84	0.93	145	23	15.9
KD-002	10	5.64	0.27	4.79	11.3	0.51	4.51	80.3	0.84	1.05	2.74	0.08	2.92	0.92	81	15.3	18.9
KD-003	10	5.6	0.1	1.79	10.3	0.2	1.94	82.7	0.25	0.30	1.36	0.03	2.21	0.96	14	2.57	18.4
NaCl-H_2O-CO_2 experiments																	
Q-011	8	5.31	0.06	1.13	9.34	0.09	0.96	80.7	0.17	0.21	4.63	0.09	1.94	0.68	189	1.49	0.79
Q-014	8	4.98	0.04	0.80	9.62	0.14	1.46	79.8	0.13	0.16	5.64	0.04	0.71	0.67	171	3.72	2.18
Q-016	7	4.93	0.07	1.42	9.64	0.11	1.14	80.1	0.25	0.31	5.3	0.11	2.08	0.69	145	3.92	2.70
Q-017	8	4.9	0.05	1.02	9.6	0.17	1.77	80.0	0.19	0.24	5.53	0.04	0.72	0.68	112	20.4	18.2
KD-005	10	5.03	0.04	0.80	9.48	0.16	1.69	80.0	0.21	0.26	5.52	0.05	0.91	0.69	142	5.22	3.68

Supplementary Table 5 Summary of experimental data and the fluid-melt partition coefficient of molybdenum.

	X_{CO2}	Salinity (NaCl. wt.%) *			C_{Mo} in fluid (leaching) §	C_{Mo} in fluid (FIs) #			C_{Mo} in Quenched melt	D_{Mo} (leaching)**	D_{Mo} (FIs) ††		
		Supercritical fluid	Brine	Vapor		Supercritical fluid	Brine	Vapor			Supercritical fluid	Brine	Vapor
H_2O-CO_2 system													
C-002	0.10	-	N.D.	N.D.	82	N.D.	N.D.	N.D.	402	0.20 (0.02)	N.D.	N.D.	N.D.
C-003	0.20	-	N.D.	N.D.	117	N.D.	N.D.	N.D.	471	0.25 (0.02)	N.D.	N.D.	N.D.
C-005	0.15	-	N.D.	N.D.	155	N.D.	N.D.	N.D.	456	0.34 (0.04)	N.D.	N.D.	N.D.
C-006	0.15	-	N.D.	N.D.	170	N.D.	N.D.	N.D.	424	0.40 (0.04)	N.D.	N.D.	N.D.
H_2O-NaCl system													
Q-006	-	1.1	N.D.	N.D.	185	239 (42.8)	N.D.	N.D.	359	0.51 (0.28)	0.66 (0.33)	N.D.	N.D.
Q-004	-	3.9	N.D.	N.D.	254	376 (140)	N.D.	N.D.	277	0.92 (0.29)	1.36 (0.53)	N.D.	N.D.
Q-001	-	6.3	N.D.	N.D.	420	402 (50.8)	N.D.	N.D.	228	1.84 (0.63)	1.77 (0.36)	N.D.	N.D.
Q-008	-	11	N.D.	N.D.	347	428 (146)	N.D.	N.D.	162	2.14 (0.99)	2.65 (1.30)	N.D.	N.D.
KD-001	-	17	N.D.	N.D.	415	461 (144)	N.D.	N.D.	145	2.86 (1.25)	3.18 (1.41)	N.D.	N.D.
KD-002	-	21	N.D.	N.D.	432	492 (75.2)	N.D.	N.D.	81.0	5.34 (2.57)	6.08 (2.48)	N.D.	N.D.
KD-003	-	44	N.D.	N.D.	290	350 (137)	N.D.	N.D.	13.6	21.3 (10.3)	25.7 (14.0)	N.D.	N.D.
H-002	-	6.6	N.D.	N.D.	435	425 (107)	N.D.	N.D.	205	2.12 (0.74)	2.07 (0.9)	N.D.	N.D.
H_2O-NaCl-CO_2 system													
		Starting salinity †											
Q-011	0.11	7.4	56	2.6	N.D.	N.D.	15425 (7750)	276 (72)	189	N.D.	N.D.	81.8 (41.1)	1.46 (0.43)
Q-014	0.15	7.8	58	N.A.	N.D.	N.D.	18455 (3094)		171	N.D.	N.D.	108 (18.7)	
Q-016	0.21	7.3	59	3.1	N.D.	N.D.	18916 (12130)	493 (65)	145	N.D.	N.D.	131 (84.1)	3.40 (1.28)
Q-017	0.31	7.1	62	N.A.	N.D.	N.D.	20023 (2722)		112	N.D.	N.D.	179 (69.7)	
KD-005	0.30	7.4	62	2.2	N.D.	N.D.	20773 (6810)	492 (72)	142	N.D.	N.D.	146 (49.1)	3.46 (1.01)

Note:

N.D. = no data.

N.A. = the data were not used because of the large uncertainty.

The data in the brackets ( ) represent 2 sigma standard deviation of the measurements.

*The salinity was determined microthermometrically and is the average for a set of fluid inclusion assemblages.

†Because of phase separation of H_2O-NaCl-CO_2 under the experimental conditions, the starting salinity of these experiments is reported.

§This concentration was measured for the quenched fluid from experiments in the H_2O-CO_2 and H_2O-NaCl systems, using a method that involved leaching of the charges with distilled water based on a modification of the method of Keppler and Willie (1991) and analysis by ICP-MS.

#This concentration represents the concentration of Mo in the reacted fluid trapped as fluid inclusions in quartz at the conditions of the experiments involving H_2O-NaCl and H_2O-NaCl-CO_2 and was analyzed by LA-ICP-MS.

**D_{Mo} in quenched melts was analyzed by LA-ICP-MS.

††D_{Mo} (leaching) was calculated from C_{Mo} in the fluid (leaching)§ divided by C_{Mo} in the quenched melt.

§§D_{Mo} (FIs) was calculated from C_{Mo} in the fluid (FIs)# divided by C_{Mo} in quenched melt.

Supplementary Table 6 Parameters and equations used in modeling the Mo extraction efficiency.

Initial Parameters	Units		Value		
Magma volume	km^3		100		
Mo concentration in magma	ppm = 10^{-6}		15		
H_2O (or fluid) content in the magma	wt%		5		
Magma density	$ton/m^3=g/cm^3$		2.5		
Vapor/brine mass ratio			10		
Equation	$M_{Mo}^{residual\ magma}=M_{Mo}^{initial\ magma}*F^{D}$ (ref. [14])				
The ratio of exsolved fluid %			The extraction efficiency %		
	D_{Mo}^{bulk} = 179*1/11+2.1*10/11		D_{Mo}^{bulk} = 81.8*1/11+1.41*10/11		$D_{Mo}^{supercritical\ fluid}$ = 1.77 (NaCl eq. = 6.3 wt.%)
	D_{Mo}^{bulk}	D_{Mo}^{bulk} calculated to peraluminium melt	D_{Mo}^{bulk}	D_{Mo}^{bulk} calculated to peraluminium melt	
0	0	0	0	0	0
2	30.7	16.8	17.1	8.4	3.51
4	52.4	31.0	31.5	16.3	6.97
6	67.5	43.0	43.6	23.6	10.4
8	78.0	53.1	53.8	30.5	13.7
10	85.3	61.6	62.3	36.8	17.0

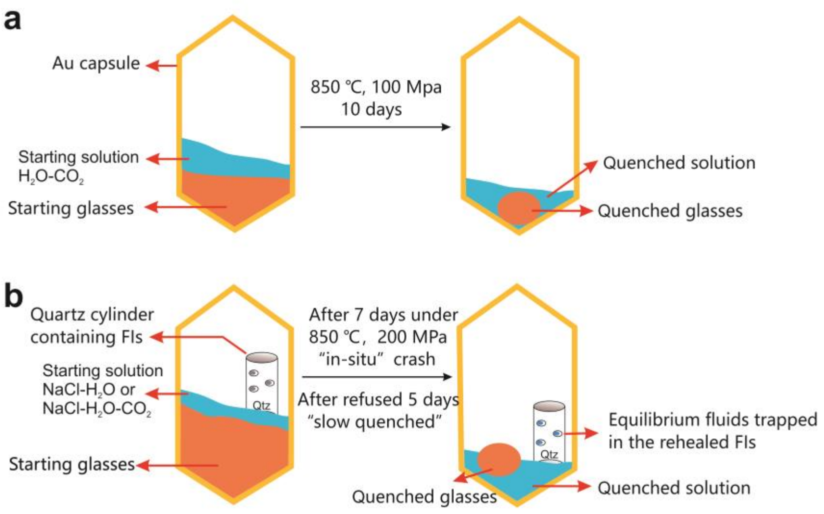

Supplementary Figure 1. Schematic illustration of the experimental design used in our experiments: **a** In the H_2O-CO_2 system, only the starting glass and solution were loaded in the gold capsule. **b** In the H_2O-NaCl and H_2O-NaCl-CO_2 systems, experiments were conducted using the previously described quartz cylinder containing Rb-bearing fluid inclusions, which were fractured in situ after the experiment had proceeded for up to 7 days. The experimental conditions were then resumed for another 5 days to ensure that the re-healed fluid inclusions trapped the fluid after equilibrium was attained.

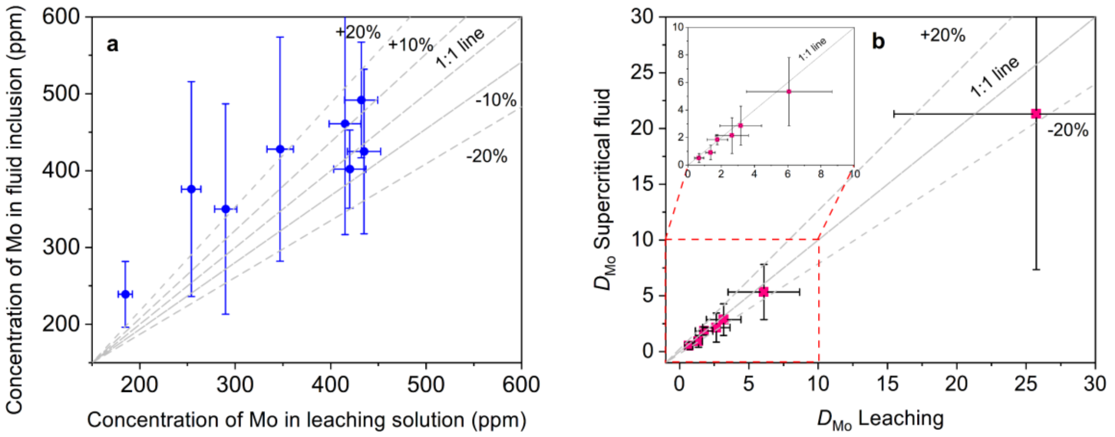

Supplementary Figure 2. A comparison of the results of the leaching and fluid inclusion methods of fluid analysis for the H_2O-NaCl experiments. The error bar illustrates 2 times standard deviation. **a** The concentration of Mo in the leaching solution analyzed by ICP-MS vs. the concentration of Mo in single fluid inclusions analyzed by LA-ICP-MS. Considering that the relative standard deviation of Mo determination in solution by ICP-MS is better than 2-4%, we adopted 4% as the uncertainty of molybdenum concentration in leaching solution measured by ICP-MS. The results demonstrate that the concentration of Mo obtained from the two method is consistent within the uncertainty; the variation between the two sets of data is mostly less than 20%. **b** A comparison of the D_{Mo} value obtained using the two methods showing that the results using the two sets of fluid composition data also very consistent.

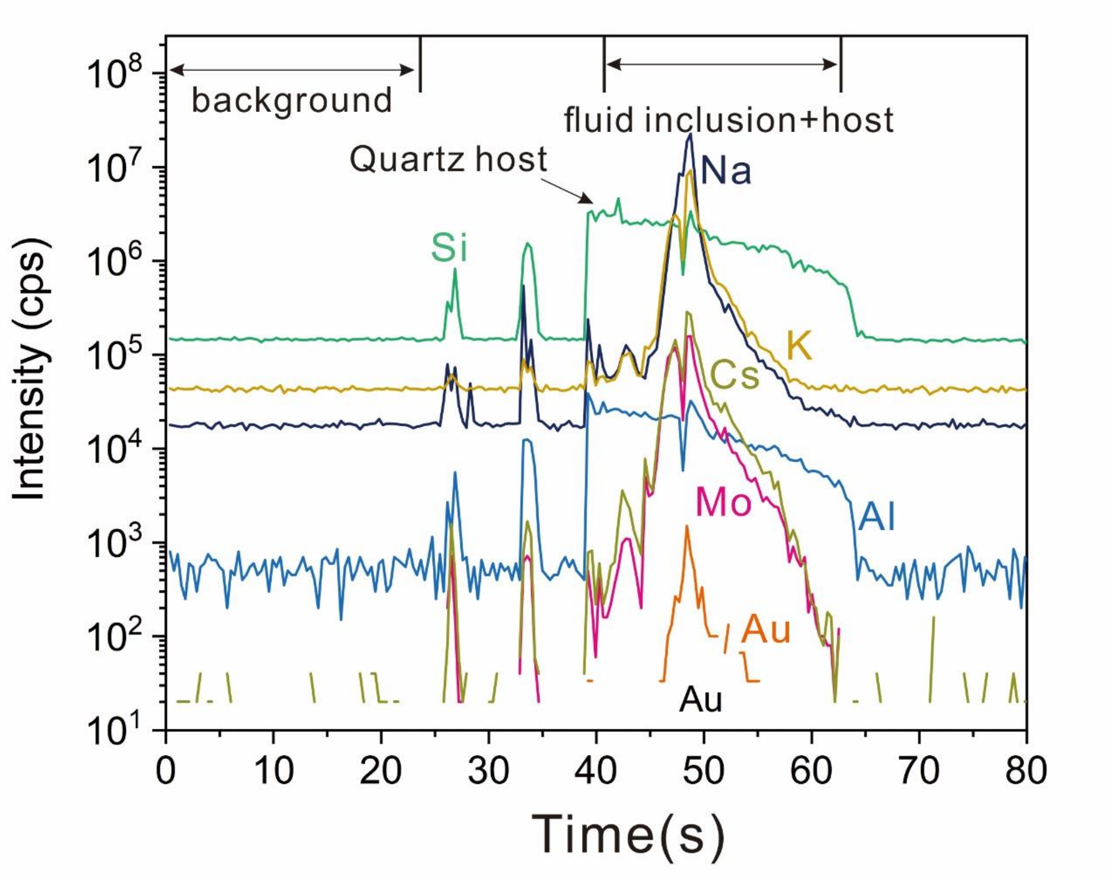

Supplementary Figure 3. The LA-ICP-MS signal obtained from a 20 μm diameter fluid inclusion trapped during experiment Q-011. The brine was trapped in the two-phase region of the $NaCl-H_2O-CO_2$ system at 850 °C, 200 MPa, and contains 15,424 ppm Mo.

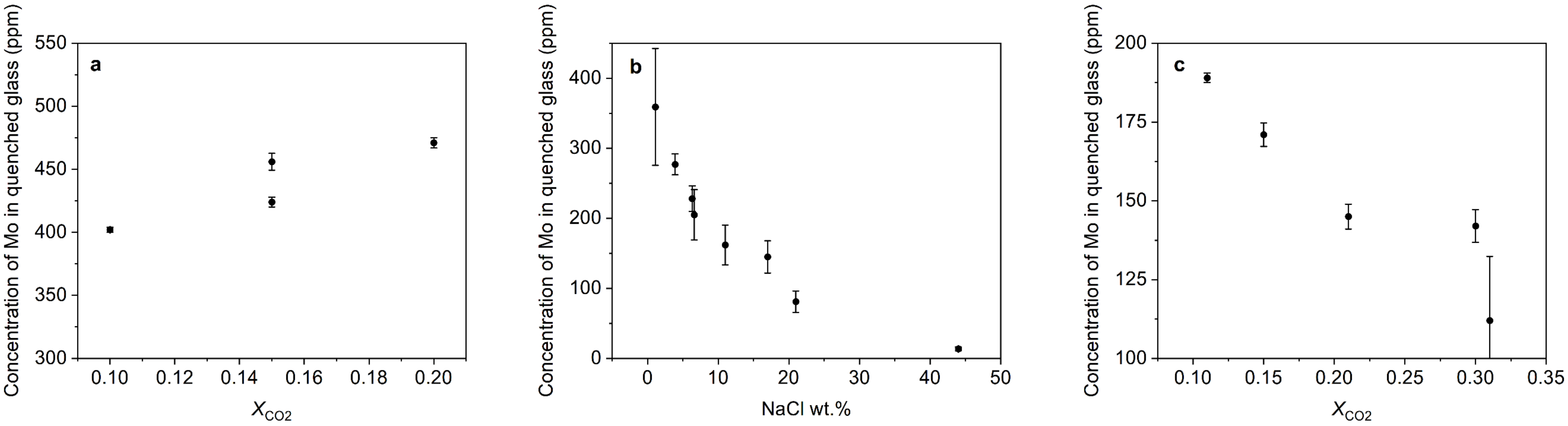

Supplementary Figure 4. Diagrams showing the variation in the concentration of Mo in the quenched glass after experiments as a function of X_{CO2} and salinity. **a** The concentration of Mo in quenched glass vs. X_{CO2} in the H_2O-CO_2 system. **b** The concentration of Mo in quenched glass vs. the salinity of the experimental solution in the H_2O-NaCl system. (**c**) The concentration of Mo in quenched glass in the H_2O-NaCl-CO_2 system. The salinity of the brine increased with increasing X_{CO2}.

Reference cited in supplemental materials.

1. Goldstein, R. H. Fluid inclusions in sedimentary and diagenetic systems. *Lithos* **55**, 159–193 (2001).

2. Candela, P. A. & Holland, H. D. The partitioning of copper and molybdenum between silicate melts and aqueous fluids. *Geochim. Cosmochim. Acta* **48**, 373–380 (1984).

3. Keppler, H. & Wyllie, P. J. Partitioning of Cu, Sn, Mo, W, U, and Th between melt and aqueous fluid in the systems haplogranite-H_2O−HCl and haplogranite-H_2O−HF. *Contrib. Mineral. Petrol.* **109**, 139–150 (1991).

4. Webster, J. D. Exsolution of magmatic volatile phases from Cl-enriched mineralizing granitic magmas and implications for ore metal transport. *Geochim. Cosmochim. Acta* **61**, 1017–1029 (1997).

5. Tattitch, B. C. & Blundy, J. D. Cu-Mo partitioning between felsic melts and saline-aqueous fluids as a function of X_{NaCleq}, fO_2, and fS_2. *Am. Mineral.* **102**, 1987–2006 (2017).

6. Fang, J. & Audétat, A. The effects of pressure, fO_2, fS_2 and melt composition on the fluid–melt partitioning of Mo: Implications for the Mo-mineralization potential of upper crustal granitic magmas. *Geochimica et Cosmochimica Acta* **336**, 1–14 (2022).

7. Zhao, P. *et al.* The partitioning behavior of Mo during magmatic fluid exsolution and its implications for Mo mineralization. *Geochimica et Cosmochimica Acta*

115–126 (2022) doi:10.1016/j.gca.2022.10.020.

8. Jiang, Z. *et al.* An experimental investigation into the partition of Mo between aqueous fluids and felsic melts: Implications for the genesis of porphyry Mo ore deposits. *Ore Geology Reviews* **134**, 104144 (2021).
9. Audétat, A. & Li, W. The genesis of Climax-type porphyry Mo deposits: Insights from fluid inclusions and melt inclusions. *Ore Geology Reviews* **88**, 436–460 (2017).
10. Chen, Y. J., Wang, P., Li, N., Yang, Y. F. & Pirajno, F. The collision-type porphyry Mo deposits in Dabie Shan, China. *Ore Geology Reviews* **81**, 405–430 (2017).
11. Li, N., Chen, Y.-J., Santosh, M. & Pirajno, F. Late Mesozoic granitoids in the Qinling Orogen, Central China, and tectonic significance. *Earth-Science Reviews* **182**, 141–173 (2018).
12. Chen, Y.J., Franco, P., Li, N., Deng, X.H. & Yang, Y.F. GEOLOGY AND GEOCHEMISTRY OF MOLYBDENUM DEPOSITS IN THE QINLING OROGEN, P R CHINA. (Springer, Singapore, 2022).
13. Wang, P. *et al.* Fluid inclusion and H–O–C isotope geochemistry of the Yaochong porphyry Mo deposit in Dabie Shan, China: a case study of porphyry systems in continental collision orogens. *Int J Earth Sci (Geol Rundsch)* **103**, 777–797 (2014).
14. Simon, A. C., Pettke, T., Candela, P. A., Piccoli, P. M. & Heinrich, C. A. Experimental determination of Au solubility in rhyolite melt and magnetite: Constraints on magmatic Au budgets. *Am. Mineral.* **88**, 1644–1651 (2003).
