# Supplementary material for: The role of CO2 in the genesis of Dabie-type porphyry molybdenum deposits
Source: Nat Commun. 2024 Jun 6;15:4849. doi: 10.1038/s41467-024-49275-0 (PMC11156875; doi:10.1038/s41467-024-49275-0)
Supplement: Supplementary file 3 — Reporting Summary [file 41467_2024_49275_MOESM3_ESM.pdf]

nature portfolio

Corresponding author(s): Lin-Bo Shang and Xin-Song Wang

Last updated by author(s): Mar 5, 2024

Reporting Summary

Nature Portfolio wishes to improve the reproducibility of the work that we publish. This form provides structure for consistency and transparency in reporting. For further information on Nature Portfolio policies, see our Editorial Policies and the Editorial Policy Checklist.

Statistics

For all statistical analyses, confirm that the following items are present in the figure legend, table legend, main text, or Methods section.

n/a	Confirmed	
☒	☐	The exact sample size (*n*) for each experimental group/condition, given as a discrete number and unit of measurement
☒	☐	A statement on whether measurements were taken from distinct samples or whether the same sample was measured repeatedly
☒	☐	The statistical test(s) used AND whether they are one- or two-sided<br>*Only common tests should be described solely by name; describe more complex techniques in the Methods section.*
☒	☐	A description of all covariates tested
☒	☐	A description of any assumptions or corrections, such as tests of normality and adjustment for multiple comparisons
☒	☐	A full description of the statistical parameters including central tendency (e.g. means) or other basic estimates (e.g. regression coefficient) AND variation (e.g. standard deviation) or associated estimates of uncertainty (e.g. confidence intervals)
☒	☐	For null hypothesis testing, the test statistic (e.g. *F*, *t*, *r*) with confidence intervals, effect sizes, degrees of freedom and *P* value noted<br>*Give P values as exact values whenever suitable.*
☒	☐	For Bayesian analysis, information on the choice of priors and Markov chain Monte Carlo settings
☒	☐	For hierarchical and complex designs, identification of the appropriate level for tests and full reporting of outcomes
☒	☐	Estimates of effect sizes (e.g. Cohen's *d*, Pearson's *r*), indicating how they were calculated

Our web collection on statistics for biologists contains articles on many of the points above.

Software and code

Policy information about availability of computer code

Data collection	No software was used.
Data analysis	No software was used.

For manuscripts utilizing custom algorithms or software that are central to the research but not yet described in published literature, software must be made available to editors and reviewers. We strongly encourage code deposition in a community repository (e.g. GitHub). See the Nature Portfolio guidelines for submitting code & software for further information.

Data

Policy information about availability of data

All manuscripts must include a data availability statement. This statement should provide the following information, where applicable:

- Accession codes, unique identifiers, or web links for publicly available datasets
- A description of any restrictions on data availability
- For clinical datasets or third party data, please ensure that the statement adheres to our policy

The authors declare that all data generated or analyzed during this study are included in this published article (and its supplementary information files). Source data are provided with this paper.

Research involving human participants, their data, or biological material

Policy information about studies with human participants or human data. See also policy information about sex, gender (identity/presentation), and sexual orientation and race, ethnicity and racism.

Reporting on sex and gender	Not applicable.
Reporting on race, ethnicity, or other socially relevant groupings	Not applicable.
Population characteristics	Not applicable.
Recruitment	Not applicable.
Ethics oversight	Not applicable.

Note that full information on the approval of the study protocol must also be provided in the manuscript.

Field-specific reporting

Please select the one below that is the best fit for your research. If you are not sure, read the appropriate sections before making your selection.

☐ Life sciences ☐ Behavioural & social sciences ☒ Ecological, evolutionary & environmental sciences

For a reference copy of the document with all sections, see nature.com/documents/nr-reporting-summary-flat.pdf

Ecological, evolutionary & environmental sciences study design

All studies must disclose on these points even when the disclosure is negative.

Study description	This study is an experimental geochemistry study. This work was not field-based, and no samples were collected from nature. We explored the role of CO2-rich ore-forming fluids in the formation of porphyry molybdenum deposits. The experiments were conducted to determine the partitioning of molybdenum between fluids in the systems H2O-CO2, H2O-NaCl, and H2O-NaCl-CO2 and felsic melt.
Research sample	No sampling from nature in our study.
Sampling strategy	No sampling from nature in our study.
Data collection	We carried out the composition analysis of the experimental products at the Institute of Geochemistry, Chinese Academy of Sciences using LA-ICP-MS and ICP-MS. The data were collected by the first author and stored in the LA-ICP-MS lab.
Timing and spatial scale	No sampling from nature in our study.
Data exclusions	This study is an experimental study. No sampling from nature. We do not exclude data unless the data is unreasonable according to the analysis methods and the rules of the LA-ICP-MS lab.
Reproducibility	All attempts to repeat the experiments were successful.
Randomization	Not relevant. This study is an experimental study. The experiments were designed to test the role of CO2 and carried out in three systems including H2O-CO2, H2O-NaCl, and H2O-NaCl-CO2.
Blinding	Not relevant. This study is an experimental study. The accuracy of the analysis were controlled by sets of standards and Quality Control standards in the LA-ICP-MS lab. The Quality Control standards acted as the role of blinding.

Did the study involve field work? ☐ Yes ☒ No

Reporting for specific materials, systems and methods

We require information from authors about some types of materials, experimental systems and methods used in many studies. Here, indicate whether each material, system or method listed is relevant to your study. If you are not sure if a list item applies to your research, read the appropriate section before selecting a response.

Materials & experimental systems

n/a	Involved in the study
☒	☐ Antibodies
☒	☐ Eukaryotic cell lines
☒	☐ Palaeontology and archaeology
☒	☐ Animals and other organisms
☒	☐ Clinical data
☒	☐ Dual use research of concern
☒	☐ Plants

Methods

n/a	Involved in the study
☒	☐ ChIP-seq
☒	☐ Flow cytometry
☒	☐ MRI-based neuroimaging

Plants

Seed stocks	Not applicable.
Novel plant genotypes	Not applicable.
Authentication	Not applicable.
